# *In vivo *^1^H-magnetic resonance spectroscopy can detect metabolic changes in APP/PS1 mice after donepezil treatment

**DOI:** 10.1186/1471-2202-10-33

**Published:** 2009-04-07

**Authors:** Eric Westman, Christian Spenger, Johanna Öberg, Henry Reyer, Jens Pahnke, Lars-Olof Wahlund

**Affiliations:** 1 Department of Neurobiology, Health Care Sciences and Society, Karolinska Institutet, Stockholm, Sweden; 2 Department of Clinical Science, Intervention and Technology, Karolinska Institutet, Stockholm, Sweden; 3 Neurological Clinic, Neurodegeneration Research Lab (NRL), Rostock, Germany

## Abstract

**Background:**

Donepezil improves cognitive functions in AD patients. Effects on the brain metabolites N-acetyl-L-aspartate, choline and *myo-*inositol levels have been reported in clinical studies using this drug. The APP/PS1 mouse coexpresses the mutated forms of human β-amyloid precursor protein (APP) and mutated human presenilin 1 (PS1). Consequently, the APP/PS1 mouse model reflects important features of the neurochemical profile in humans. *In vivo *magnetic resonance spectroscopy (^1^H-MRS) was performed in fronto-parietal cortex and hippocampus (ctx/hipp) and in striatum (str). Metabolites were quantified using the LCModel and the final analysis was done using multivariate data analysis. The aim of this study was to investigate if multivariate data analysis could detect changes in the pattern of the metabolic profile after donepezil treatment.

**Results:**

Significant differences were observed in the metabolic pattern of APP/PS1 mice in both str and ctx/hipp before and after donepezil treatment using multivariate data analysis, evidencing a significant treatment effect. A treatment effect was also seen in wild type (wt) mice in str. A significant decrease in the metabolic ratio taurine/creatine (Tau/tCr) was related to donepezil treatment (p < 0.05) in APP/PS1 mice in both brain regions. Furthermore, a significant influence on the choline/creatine (tCho/tCr) level was observed in treated APP/PS1 mice compared to untreated in str (p = 0.011). Finally, there was an increase in glutamate/creatine (Glu/tCr) in str in wt mice treated with donepezil.

**Conclusion:**

Multivariate data analysis can detect changes in the metabolic profile in APP/PS1 mice after donepezil treatment. Effects on several metabolites that are measurable *in vivo *using MR spectroscopy were observed. Changes in Tau/tCr and tCho/tCr could possibly be related to changed cholinergic activity caused by donepezil treatment.

## Background

Proton magnetic resonance spectroscopy (^1^H-MRS) provides useful information on the neurochemical profile in different neurodegenerative diseases [1,2]. It is a non-invasive method which allows the investigation of the chemical profile of defined target volumes in subjects *in vivo*. Examples of measurable metabolites are N-acetylaspartate (NAA) a marker for neuronal density and/or function, myo-inositol (m-Ins) a marker for astrogliosis and/or osmotic stress and choline (Cho) a marker for cell membrane turnover and degradation [3]. Brain metabolites are sensitive to pathological processes in neurodegenerative disorders such as Alzheimer's disease (AD) [2].

AD is one of the most common forms of neurodegenerative disorders [4]. The clinical symptoms of AD include progressive memory impairment, disordered cognitive functions, altered behaviour including paranoia, delusions, loss of social appropriateness and decline in language functions. The majority of the prevalent AD cases are sporadic. The small percentage of remaining AD cases are familial and connected to specific gene mutations [4].

There are few approved treatment options for AD. One of them is the administration of acetylcholine-esterase (AchE) inhibitors, like donepezil which enhances the life of the neurotransmitter acetylcholine in the synapse and thus increases the cholinergic neurotransmission [5]. Symptomatically, donepezil improves cognitive functions in AD patients[6,7]. Using Magnetic Resonance spectroscopy (MRS) in patients, brain metabolites and neurotransmitters can be non-invasively measured and effects on brain NAA levels caused by donepezil have been reported [7,8]. Moreover, effects on m-Ins and Cho in hippocampus of AD patients treated with donepezil have been observed [9].

Different animal models that express one or more mutant proteins associated with AD allow us to study different features of AD [10-14]. While some models may express different levels of β-amyloid [15,16], other models modulate the production of tangles [17,18]. We investigated the neurochemical profile of the double transgenic (tg) APP/PS1 mouse [19,20] during maturation and ageing in a previous study [21]. As a continuation of this, we have investigated the effects of donepezil treatment in the same mouse model. The APP/PS1 mouse coexpresses the mutated forms of human β-amyloid precursor protein (APP) and mutated human presenilin 1 (PS1) and thus reflects important features of the neurochemical profile in humans [22].

The aim of this study was to investigate the possibility of monitoring early donepezil treatment effects in young APP/PS1 mice with the combination of multivariate data analysis and MRS. By doing so we wanted to observe changes in the metabolic pattern as well as in individual metabolites. We did not expect significant changes in the levels of NAA and m-Ins as previously reported in humans treated with donepezil. We based this on the fact that these metabolites are not altered in the APP/PS1 mice model until later in their development. Our hypothesis was based on the multivariate model looking at the underlying metabolic pattern rather than individual metabolites. However, we believed the levels of choline were most likely to be altered, since choline is a precursor of acetylcholine and donepezil is an inhibitor of acetylcholine-esterase.

## Results

### Magnetic resonance spectroscopy (^1^H-MRS)

**Figure 1:**
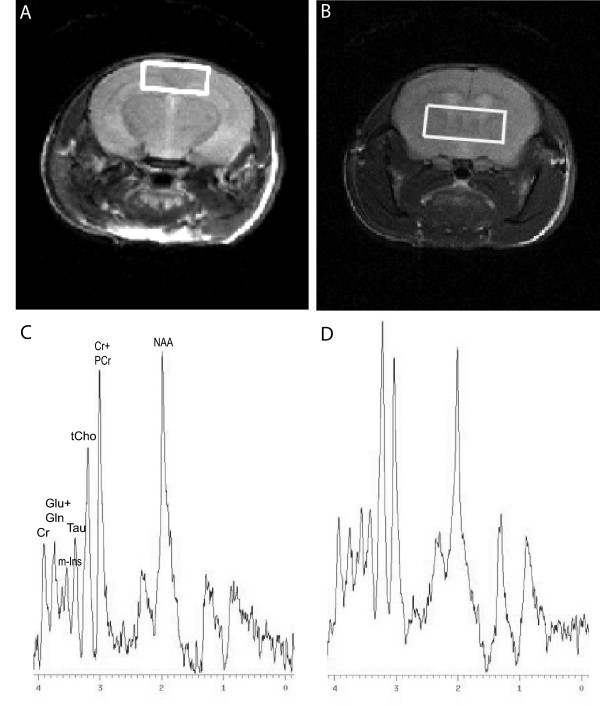
**MRS voxel placement and spectrum.** (A) Representative MRI featuring a frontal slice through a mouse brain with localization 1.5 mm posterior to bregma in cortex/hippocampus. Placement of the voxel of interest (VOI) sized 4.0 × 1.6 × 1.6 mm^3 ^for spectroscopy as indicated by the white box. (B) Representative MRI featuring a frontal slice through a mouse brain with localization 0.5 mm anterior to bregma in striatum. Placement of the VOI sized 5.0 × 2.0 × 2.0 mm^3 ^for spectroscopy as indicated by the white box. (C) Representative wild type spectra from cortex/hippocampus to show the quality of spectra received. (D) Representative wild type spectra from striatum to show the quality of spectra received.

^1^H-MRS was used to monitor differences in metabolite contents *in vivo *before and after donepezil treatment in APP/PS1 mice. Also wt mice were investigated before and after treatment. Two different volumes of interest (VOI) were used in this study. The first one included parietal cortex and hippocampus (10.24 mm^3^) and the second VOI was placed in striatum (20 mm^3^). Fig. 1A (ctx/hipp) and 1B (str) demonstrates the placement of the two different volumes of interest. A representative spectrum from ctx/hipp of a wt mouse is shown in Fig. 1C and a representative spectrum from str is shown in Fig. 1D. Six different variables (metabolite ratios) were quantified using the software LCModel. These were tNAA/tCr, tCho/tCr, Tau/tCr, m-Ins/tCr, Glu/tCr and Gln/tCr. Mean metabolic ratios for all groups are shown in table 1.

**Table 1:** Mean metabolic ratios in each animal group before and after donepezil and NaCl respectively.

**Mice type**	**Brain region**	**Treatment**	**Gln/tCr**	**Glu/tCr**	**m-Ins/tCr**	**Tau/tCr**	**tCho/tCr**	**tNAA/tCr**
			**Mean ± SD**					

tg	str	**before**	0.64 ± 0.11	1.30 ± 0.13	0.96 ± 0.11	1.28 ± 0.10	0.33 ± 0.03	0.90 ± 0.13
tg	str	donepezil	0.77 ± 0.28	1.27 ± 0.34	0.89 ± 0.11	0.97 ± 0.23	0.38 ± 0.01	0.93 ± 0.15
tg	str	NaCl	0.59 ± 0.07	1.47 ± 0.22	0.97 ± 0.13	1.29 ± 0.14	0.39 ± 0.03	0.99 ± 0.07
tg	ctx/hipp	**before**	0.61 ± 0.13	1.48 ± 0.26	0.62 ± 0.15	0.86 ± 0.10	0.24 ± 0.03	1.19 ± 0.11
tg	ctx/hipp	donepezil	0.72 ± 0.08	1.60 ± 0.30	0.57 ± 0.07	0.73 ± 0.07	0.24 ± 0.03	1.24 ± 0.20
tg	ctx/hipp	NaCl	0.72 ± 0.17	1.60 ± 0.23	0.55 ± 0.14	0.85 ± 0.13	0.25 ± 0.04	1.18 ± 0.21
wt	str	**before**	0.61 ± 0.14	1.18 ± 0.12	0.78 ± 0.09	1.14 ± 0.10	0.34 ± 0.02	0.88 ± 0.10
wt	str	donepezil	0.67 ± 0.11	1.34 ± 0.16	0.79 ± 0.12	1.22 ± 0.13	0.36 ± 0.02	0.93 ± 0.05
wt	str	NaCl	0.72 ± 0.10	1.25 ± 0.11	0.78 ± 0.09	1.21 ± 0.13	0.34 ± 0.03	0.90 ± 0.06
wt	ctx/hipp	**before**	0.46 ± 0.16	1.37 ± 0.14	0.52 ± 0.08	0.78 ± 0.08	0.22 ± 0.02	0.96 ± 0.09
wt	ctx/hipp	donepezil	0.49 ± 0.09	1.34 ± 0.11	0.52 ± 0.06	0.75 ± 0.09	0.24 ± 0.03	0.99 ± 0.11
wt	ctx/hipp	NaCl	0.46 ± 0.06	1.41 ± 0.20	0.52 ± 0.09	0.77 ± 0.09	0.22 ± 0.02	1.03 ± 0.10

Abbreviations: Gln = Glutamine, Glu = Glutamate, Tau = Taurine, m-Ins= Myo-inositol, tCho = Choline-containing compounds, tNAA = N-acetylaspartate+N-acetylaspartylglutamate, wt = wild type mice, tg = APP/PS1 transgenic mice, ctx/hipp = parietal cortex/hippocampus, str = striatum.

### Donepezil treatment effects on cortex/hippocampus depicted by multivariate data analysis

**Figure 2:**
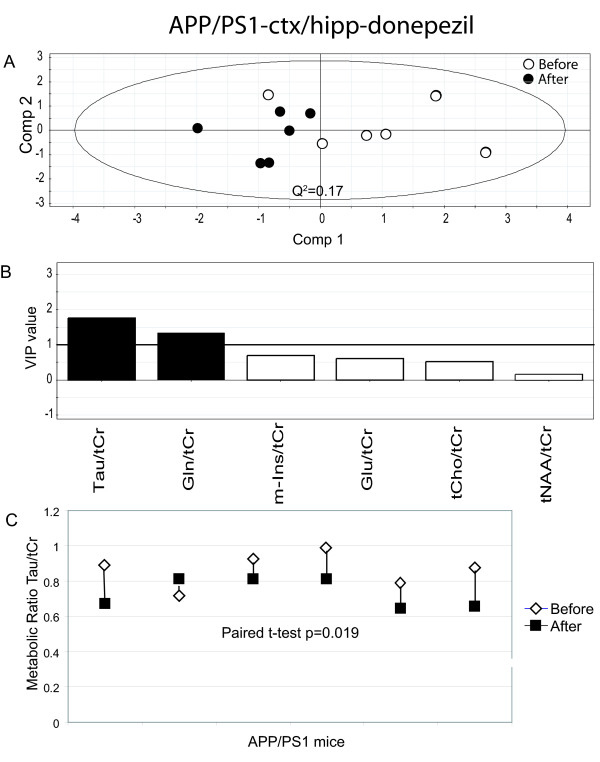
**Results from the data analysis of donepezil treated APP/PS1 (cortex/hippocampus).** (A) PLS-DA scatter plot which displays a clear separation between mice before and after donepezil treatment (white circles = before treatment and black circles after treatment). Each data point represents one mouse. A Q^2^-value above 0.05 indicates that the groups can be significantly distinguished along that component. (B) Metabolites which are important for the separation between groups in the PLS-DA model. VIP (variable of importance in the projection) values larger than 1 are relevant in explaining group differences (black bars) while metabolites with values below 1 have no significant impact (white bars). (C) The paired t-test shows a significant decrease in Tau, p = 0.019. White diamonds represent before treatment and black squares after treatment.

Multivariate data analysis was used to compare differences between mice before and after treatment. First we compared spectra from ctx/hipp in APP/PS1 mice. Fig. 2A shows a scatter plot of the PLS-DA group comparison between APP/PS1 mice before and after treatment. A significant separation (Q2 = 0.17) between the groups can be observed. The first principle component (comp 1) describes 49% of the variance of the original data. Leave-one-out cross validation predicted 6/6 treated animals and 5/6 untreated animals correctly (specificity = 100%; sensitivity = 83%). There were no effects on the brain metabolites in the non-treated APP/PS1 mice. Moreover, no effect was observed on the metabolites in ctx/hipp in donepezil or untreated wt mice (not shown).

### Donepezil treatment effects on striatum depicted by multivariate data analysis

**Figure 3:**
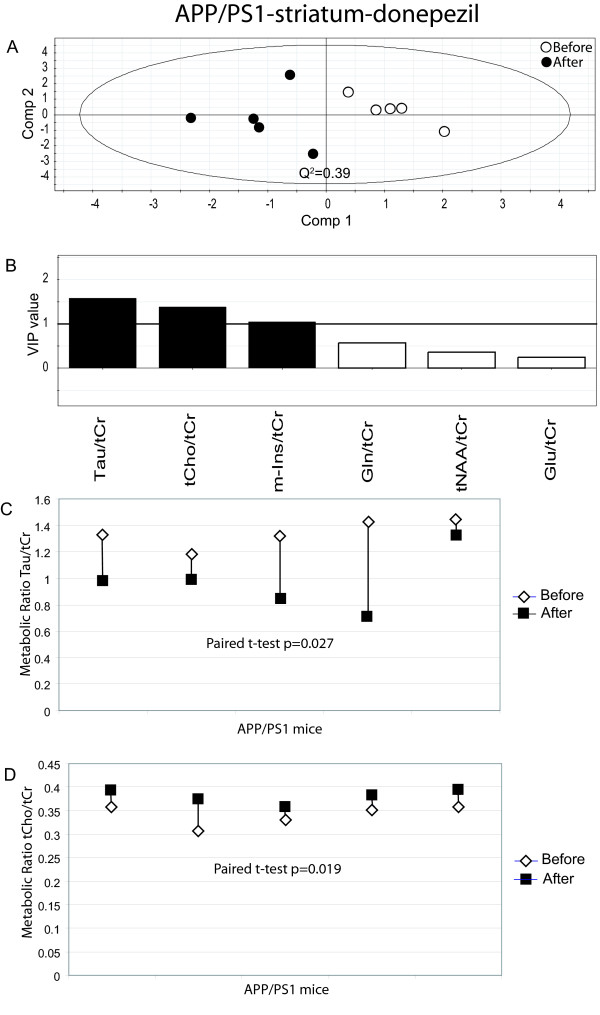
**Results from the data analysis of donepezil treated APP/PS1 (striatum)**. (A) PLS-DA scatter plot which displays a clear separation between mice before and after donepezil treatment (white circles = before treatment and black circles after treatment). Each data point represents one mouse. A Q^2^-value above 0.05 indicates that the groups can be significantly distinguished along that component. (B) Metabolites which are important for the separation between groups in the PLS-DA model. VIP (variable of importance in the projection) values larger than 1 are relevant in explaining group differences (black bars) while metabolites with values below 1 have no significant impact (white bars). (C) The paired t-test shows a significant decrease in Tau, p = 0.027. White diamonds represents before treatment and black squares after treatment. (D) The paired t-test shows a significant increase in tCho, p = 0.019. White diamonds represent before treatment and black squares after treatment.

**Figure 4:**
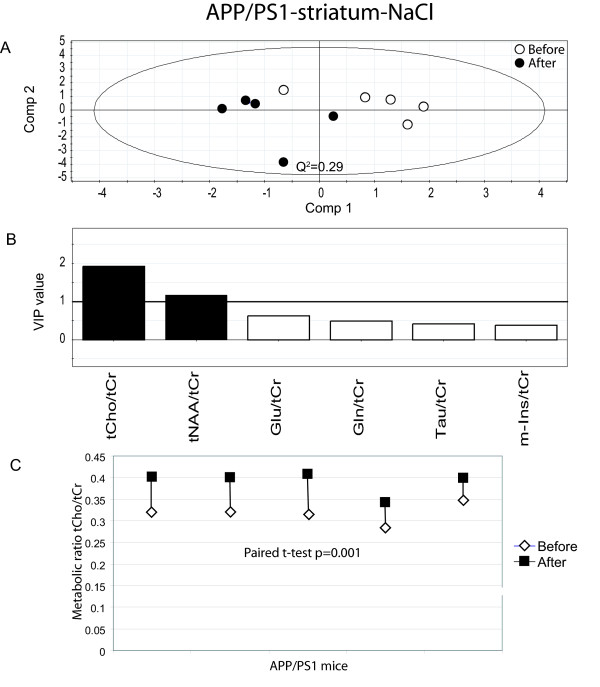
**Result from the data analysis of saline treated APP/PS1 (striatum)**. (A) PLS-DA scatter plot which displays a clear separation between mice before and after saline treatment (white circles = before treatment and black circles after treatment). Each data point represents one mouse. A Q^2^-value above 0.05 indicates that the groups can be significantly distinguished along that component. (B) Metabolites which are important for the separation between groups in the PLS-DA model. VIP (variable of importance in the projection) values larger than 1 are relevant in explaining group differences (black bars) while metabolites with values below 1 have no significant impact (white bars). (C) The paired t-test shows a significant increase in tCho, p = 0.001. White diamonds represent before treatment and black squares after treatment.

We then investigated spectra from str in APP/PS1 and wt mice before and after treatment. There is a clear separation between APP/PS1 animals before and after treatment with donepezil (Fig. 3A.). There is one significant (Q2 = 0.39) component that explains 75% of the variance of the original data. Leave-one-out cross validation predicted 5/5 treated animals and 5/5 untreated animals correctly (specificity = 100%; sensitivity = 100%). Interestingly, we also noted a separation between the spectra targeted in str of untreated APP/PS1 mice (Fig. 4A). The first principle component (Q2 = 0.29) explains 58% of the variance of the original data. Leave-one-out cross validation predicted 4/5 treated animals and 4/5 untreated animals correctly (specificity = 80%; sensitivity = 80%).

**Figure 5:**
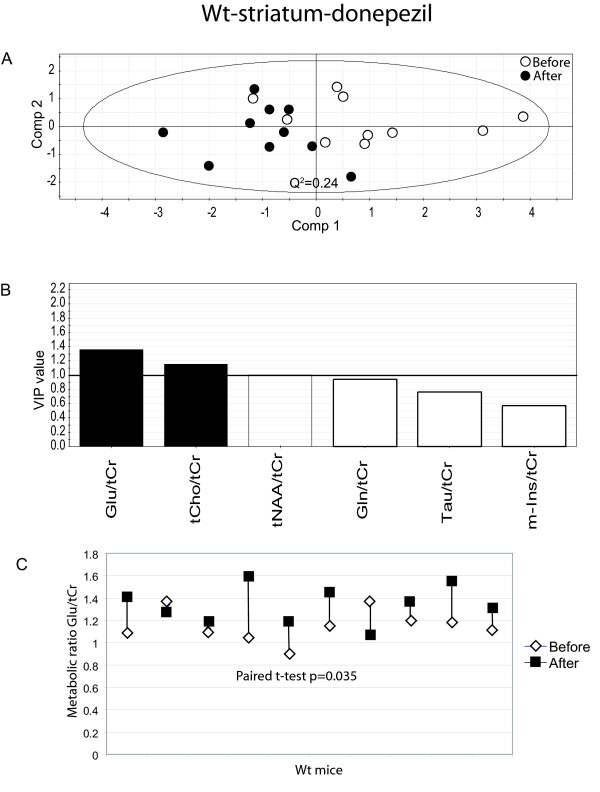
**Results from the data analysis of donepezil treated wild type mice (striatum)**. (A) PLS-DA scatter plot which displays a clear separation between mice before and after donepezil treatment (white circles = before treatment and black circles after treatment). Each data point represents one mouse. A Q^2^-value above 0.05 indicates that the groups can be significantly distinguished along that component. (B) Metabolites which are important for the separation between groups in the PLS-DA model. VIP (variable of importance in the projection) values larger than 1 are relevant in explaining group differences (black bars) while metabolites with values below 1 have no significant impact (white bars). (C) The paired t-test shows a significant increase in Glu, p = 0.035. White diamonds represent before treatment and black squares after treatment.

A treatment effect of donepezil was also observed in str for wt mice (Fig. 5A). One significant component is seen (Q2 = 0.24) to explain 38% of the variance of the original data and leave-one-out cross validation predicted 9/10 treated animals and 8/10 untreated animals correctly (specificity = 90%; sensitivity = 80%). No effect in str of wt animals receiving saline injections was observed (not shown).

### Metabolite ratios of importance for separation between groups

PLS-DA modelling pinpoints metabolite ratios which are responsible for the separation between groups. Metabolite ratios with a variable of importance (VIP) value larger than one are over average responsible for the differences. Hence, it can be seen that the most important metabolite ratios for the separation of APP/PS1 mice before and after donepezil treatment were Tau/tCr and Gln/tCr for measurements in ctx/hipp (Fig. 2B) and Tau/tCr, tCho/tCr, m-Ins/tCr for measurement in str (Fig. 3B). Interestingly, there were also differences in the metabolic fingerprint in untreated APP/PS1 mice in str. This might indicate changes caused by disease progress. The metabolite ratios that were responsible for these differences were tCho/tCr and tNAA/tCr (Fig. 4B). Moreover, metabolite ratios of importance for the separation for treated wt mice in str (Fig. 5B) were Glu/tCr and tCho/tCr.

All individual metabolite ratios of importance for the separations between different groups were tested for their statistical significance using the paired t-test. As depicted in Fig 2C, Tau/tCr was significantly decreased in ctx/hipp in APP/PS1 mice during the four weeks of treatment (p = 0.019). However, there was no statistically significant difference for Gln/tCr after treatment in these mice. Moreover, for APP/PS1 mice a significant decrease in Tau/tCr (p = 0.027) in str (Fig. 3C) was seen, which is in line with the measurements from ctx/hipp. The metabolite ratio tCho/tCr was significantly increased in str (Fig. 3D) in APP/PS1 mice (p = 0.019) after donepezil treatment. The metabolite tCho/tCr was also significantly increased in str in the untreated APP/PS1 mice (p = 0.001) (Fig. 4C). As can be observed when comparing Fig. 3D and Fig. 4C the increase in tCho/tCr is smaller in donepezil treated than in untreated APP/PS1 mice. This was statistically verified using a two-sample t-test (p = 0.011). The other metabolite ratios of importance for the separation between treated and untreated animals for APP/PS1 mice did not reach statistical significance on an individual level. For wt mice the only metabolite ratio reaching statistical significance was Glu/tCr (p = 0.035) (Fig. 5C).

### Acetylcholine-esterase (AchE) activity

To test the effect of donepezil as an acetylcholine-esterase inhibitor, the brains of wt and tg mice were analyzed for acetylcholine-esterase activity at the end of the treatment (Table 2). There was a significant difference between treated and non treated wt mice (p = 0.007). In the APP/PS1 mice the mean activity in donepezil treated animals was seemingly lower than the mean of animals receiving NaCl injections; however it did not reach statistical significance.

**Table 2:** AchE activity.

**Mouse type**	n	**Treatment**	**Mean ± SD**
tg	5	donepezil	0.052 ± 0.005
tg	5	NaCl	0.056 ± 0.007
wt	10	donepezil	0.049 ± 0.005
wt	10	NaCl	0.056 ± 0.005

Abbreviations: wt = wild type mice, tg = APP/PS1

## Discussion

### Multivariate data analysis

We have demonstrated that by employing a combination of ^1^H-MRS and multivariate data analysis, it is possible to observe a treatment effect of donepezil in parietal cortex/hippocampus and in striatum of APP/PS1 mice. We also found a treatment effect in wt mice in str, but not in ctx/hipp. Interestingly, the Tau/tCr ratio was decreased in str and ctx/hipp of APP/PS1 mice due to donepezil treatment. We also found a less pronounced increase in tCho/tCr in str for treated APP/PS1 mice compared to untreated.

This shows that multivariate data analysis is a powerful tool in analyzing complex biological data and that it is possible to monitor treatment effects at an early stage. The strength of multivariate data analysis compared to traditional univariate data analysis methods, such as the t-test, is that it correlates the different metabolites to each other and considers them all simultaneously. By doing so, it is possible to observe patterns in the data not possible when looking at the metabolites one by one.

The most significant separation between groups before and after treatment was seen in str in APP/PS1 mice. A significant separation, although less pronounced, was also seen in ctx/hipp of APP/PS1 mice. Different density and levels of expression of AchE in ctx/hipp and str may partly explain this variance. In line with this, we saw a significant separation between wt mice before and after treatment of donepezil in str but not in ctx/hipp.

### Tau/tCr decrease in donepezil treated APP/PS1 mice in parietal cortex/hippocampus and striatum

Tau is a free amino acid present in the brain. Areas in the brain which contain the highest concentrations of Tau are dorsal striatum and hippocampus [23]. This metabolite is reported to be involved in a number of biological functions. It acts as an organic osmolyte in the brain, involved in cell volume regulation. Another role of taurine is modulation of the actions of neurotransmitters. The concentration of Tau is much higher in rodent brains than in human brains [24]. Dedeoglu et al showed an increase in Tau in aged APP transgenic mice (age 20 months). Since Tau plays a role in osmoregulation, similar to the role of m-Ins in humans, this increase is suggested to reflect a similar process to that which increased m-Ins plays in human AD [24]. Marijanska et al showed an increase in m-Ins in APP/PS1 mice (aged 20 months) and no increase in Tau suggesting that this mouse model most closely matches the neurochemical profile of human AD [22]. When we investigated APP/PS1 mice aged 2.5 to 9.0 months, we found no increase in Tau/tCr nor in m-Ins/tCr in APP/PS1 mice [21]. Since no change of Tau/tCr in APP/PS1 mice was detected within the first 9 months of life, the observed decrease in Tau/tCr in the present study indicates that it is a direct effect of donepezil treatment. This is supported by the fact that the decrease in Tau/tCr was observed in both ctx/hipp and str, which are both brain regions with high concentrations of Tau. Also no effect on Tau/tCr was seen in untreated APP/PS1 mice. Finally, no decrease in Tau/tCr was seen in wt mice receiving donepezil. It has previously been shown in rats that levels of the amino-acid Tau are lowered following treatment with another AchE inhibitor (ENA713) [25]. It was further suggested that the AchE inhibitor, metrifonate has additional properties which modulate other neurochemical systems than the cholinergic [26].

The decrease in Tau may be directly connected to the cholinergic activity, which is the target of many anti-AD drugs. It has been shown that Tau inhibits the release of Ach [27]. This suggests that lower levels of Tau lead to a reduced inhibitory tone, resulting in increased cholinergic activity [25].

### Progressive increase in striatal tCho/Cr of APP/PS1 mice is partially mitigated by donepezil treatment

We observed a significant increase in striatal tCho/tCr of APP/PS1 mice. However, this increase was significantly lower in mice receiving donepezil compared to mice receiving saline. We interpret the observed increase in the levels of tCho/tCr in striatum of APP/PS1 mice to be an effect of the disease process. This is in line with human patient studies, where elevations in choline concentrations have also been measured with MRS [2,28-30]. The increase we observed is less pronounced in the animals receiving donepezil treatment, which is in line with a recent MRS study demonstrating a decrease in tCho levels in probable AD patients treated with donepezil [9]. In this study Bartha et al. suggest that the change in tCho they observed in AD patients could reflect a decrease in phosphatidylcholine catabolism leading to lower levels of tCho containing compounds, which are required precursors for acetylcholine synthesis. The substances that mainly contribute to the choline peak in a MRS spectrum are free choline, glycerophosphocholine and phosphocholine [28]. Choline is a precursor of acetylcholine and it is also a product of membranial phosphatidylcholine breakdown [1]. In AD there are losses of cholinergic neurons and it was shown in vitro that levels of free choline and glycerophosphorylcholine are higher in AD brains [31]. Moreover, higher levels of choline may be explained by an increased cell membrane phospholipid turnover in demented brains [28]

The target of donepezil is to inhibit the acetylcholine breakdown by decreasing cholinesterase activity. Our results are in full agreement with the studies discussed above and they demonstrate a progressive increase in striatal tCho/Cr, which is partially mitigated by donepezil treatment in this mouse model of Alzheimer's disease.

### Glu/tCr increase in donepezil treated wt mice in striatum

Glutamate is the most abundant neurotransmitter in the brain. There is no specific theory on the role of glutamate in AD. In general a decrease in glutamate is suggested to be due to the death of glutamatergic cortical neurons and an increase may reflect a potentially neurotoxic effect. Glutamate also has a role in synaptic plasticity and because of this it is believed that glutamic acid is involved in learning and memory. We observed an increase in the Glu/tCr ratio in the striatum of wt mice after donepezil treatment. This finding is somewhat unexpected and at present we have no plausible explanation. These changes in glutamate might be connected to increased cholinergic activity but we cannot rule out the possibility of direct effects on glutamate levels from donepezil. Further studies are required to investigate these findings.

### Acetylcholine-esterase (AchE) activity

Donepezil significantly decreased the acetylcholine-esterase activity in wt mice, which might explain the increased levels of Glu/tCr due to increased cholinergic activity. However, donepezil did not significantly decrease the acetylcholine-esterase activity in APP/PS1 mice. There are several different explanations for this. One of the reasons could be the small number of APP/PS1 mice tested compared to wt mice. Another reason is the dose of donepezil used. In order to confirm that the right dose was used, a dose response study is warranted.

## Conclusion

Treatment effects of the acetylcholine-esterase inhibitor, donepezil can be detected in str and ctx/hipp of APP/PS1 mice by analyzing *in vivo *MRS data with multivariate data analysis (PLS-DA). A disease dependent tCho/tCr increase was observed in str of APP/PS1 mice. This increase was significantly lower in APP/PS1 mice receiving donepezil, which reflects a reversal of the levels of this metabolite ratio. Moreover we found a decrease in Tau/tCr level in both str and ctx/hipp s in APP/PS1 mice as a result of donepezil treatment. Both the decrease in Tau/tCr and the less pronounced increase in the tCho/tCr level might possibly be mediated by changed cholinergic activity as a result of the donepezil treatment.

## Methods

### Experimental animals

23 female mice B6C3-Tg(APPswe, PSEN1dE9)85Dbo/JTg/0 from The Jackson Laboratory (Bar Harbor, ME, USA) and 20 female mice C57BL/6/Sca (wt) from Scanbur BK, Denmark were used. The studies were approved by the Northern Stockholm Ethics Committee on Experimental Animal Care and performed in accordance with the guidelines from the Swedish National Board for Laboratory Animals. Table 3 shows the different mouse groups, age, number of animals, treatment and brain region used for MRS.

**Table 3:** Mice groups.

**Mouse type**	**n**	**Age at beginning of treatment**	**Treatment**	**Investigated brain region**
tg	6	12 weeks	donepezil	ctx/hipp
tg	7	12 weeks	NaCl	ctx/hipp
tg	5	16 weeks	donepezil	str
tg	5	16 weeks	NaCl	str
wt	10	12 weeks	donepezil	ctx/hipp and str
wt	10	12 weeks	NaCl	ctx/hipp and str

Abbreviations: wt = wild type mice, tg = APP/PS1transgenic mice, ctx/hipp = parietal cortex/hippocampus, str = striatum.

### Treatment

The mice were kept in a 12 h light/dark circle. Rodent breeding diet R36 (Lactamin AB, Stockholm, Sweden) and water were provided ad libitum. Donepezil (Eisai, Ibaraki, Japan) was administrated i.p. daily for four weeks (0.6 mg/kg, i.p.) from postnatal week 12 to 16 or from 16 to 20. Mice not treated with Donepezil received i.p. saline injection daily for four weeks (0.9% NaCl, i.p.). The mice showed no sign of toxicity (i.e. no decreased locomotion, drowsiness, or loss of weight).

### Acetylcholine-esterase (AchE) activity assay

The protocol was adapted from [32,33]. Chemicals were purchased from Sigma or Roth if not otherwise specified. Frozen brain hemispheres were homogenized in a 1.5 mL homogenization buffer (1 mM Sodiumhydrogencarbonate, 0.2 mM Magnesiumchloride, 0.2 mM Calciumchloride, 1 mM Spermidine, adjusted to pH 8.0). The protein concentration was determined using the BCA assay (Pierce). Prior to the measurements, samples were diluted 1:2 in the homogenization buffer and incubated as described in the assay description. Measurements were made using a TecanGenios ELISA reader (Tecan, Crailsheim, Germany). Samples were then adjusted to 1 μg/μL protein contents using the homogenization buffer. 21 μL of the adjusted samples were then incubated in the following buffers: a) 294 μL of 0.1 M Potassiumphosphate (pH 8.0); b) 21 μL DTNB solution (10 mM 5,5'-Dithio-bis-2-nitro-benzoic acid in 0.1 MKPi pH 8.0 (titrated from 1 M potassiumphosphate and 1 M potassiumhydrogenphosphate) supplemented with 100 mg sodiumhydrogencarbonate/100 mlKPi). The solution was then gently mixed for 3 min. at room temperature and finally 14 μL of the substrate 75 mM acetylthiocholine-iodid (Fluka) was added to start the reaction. The primary mix was substituted by 0.1 mM AchE inhibitor BW284C51 for the negative controls. The measurements were carried out in 60s time steps using the TecanGenios ELISA Reader at 412 nm.

### Animal preparation and anaesthesia

All mice were subjected to a MRI/^1^H-MRS investigation at the beginning and the end of the 4 week donepezil or saline treatment. The mice were anesthetized with 1.5–2.0% isoflurane delivered through a face mask, allowing for spontaneous respiration. The mouse was positioned in supine position and the head fixed to the acrylic rig. Body temperature was recorded and maintained at 36 to 37°C using a MRI-compatible air temperature control system. Respiratory rate was monitored continuously (BioTrigger, Bruker, Karlsruhe, Germany).

### MRI and ^1^H-MRS

MRI examinations were performed using a 4.7 T magnet with a horizontal bore (Bruker Biospec Avance 47/40, Bruker, Karlsruhe, Germany) equipped with a 12 cm inner diameter self-shielded gradient system (max. gradient strength 200 mTm^-1^). A commercially available volume coil (Bruker, Karlsruhe, Germany) with an inner diameter of 25 mm was used for excitation and signal detection.

Volumes of interest for spectroscopy were localized using spin echo sequences with rapid acquisition with relaxation enhancement (RARE) imaging [34] producing either a 3D volume with 64 × 64 phase steps covering the whole brain or 2D slices in axial, coronal and sagital direction consisting of 11, 7 or 9 continuous slices, respectively. The parameters for 3-D inversion recovery were adjusted as follows: repetition time (TR) 2566.8 ms, echo time (TE) 35.6 ms, RARE-factor 8 with RARE-maximum 4, inversion time 450 ms, matrix size 64 × 64 × 128 and 2 averages. The 3D volume measured 0.90 × 1.20 × 1.80 cm. The parameters for the 2-D slices were adjusted as follows: TR 3000 ms, TE 37.4, RARE-factor 8 with RARE-maximum 4, matrix size 256 × 256, slice thickness 1 mm, FOV 2.5 mm, 8 averages. The volume containing 11 consecutive 2D slices in the axial direction was positioned with the first slice at the rhinal fissure.

In this study single voxel ^1^H-MRS was performed in striatum (str) and in parietal cortex/hippocampus (ctx/hipp) in APP/PS1 and wild type (wt) mice. Braak and Braak (1991) have described the progression of AD. It evidently progresses from etorhinal cortex and hippocampus. The pathology then continues to parietal cortex and frontal cortex, before finally reaching the entire brain. We chose to place the voxel of interest for the MRS experiment in parietal cortex/hippocampus, due to the fact that AD pathology starts there. Our MRS measurements in parietal cortex/hippocampus showed effects on the brain metabolite ratio taurine/creatine. Taurine is a metabolite which can be found in high concentrations in striatum as well as in hippocampus [23]. Therefore, we placed the second MRS voxel in striatum. Great care was taken to position the voxel in the same location for each animal. The voxels were placed measuring from the rhinal fissure, where the first 2D slice had been positioned to the pre-assigned VOI location. The voxel in ctx/hipp (Figure 1A) was placed 1.5 mm posterior to bregma and 0.5 mm anterior to bregma in str (Figure 1B). The VOI size was 4.0 × 1.6 × 1.6 mm and represented a volume of 10.24 mm^3 ^in ctx/hipp and 5.0 × 2.0 × 2.0 mm resulting in a volume of 20 mm^3 ^in str. A PRESS sequence with the following parameters was used for MRS: TR 3500 ms, TE 20 ms, VAPOR water suppression [35], 512 averages in ctx/hipp and 256 averages in str. The line width of the water peak was assessed in the VOI before each MRS experiment to check the shim quality. This was performed after adjusting the first order shims using an unsuppressed water scan of 1 average. Resulting line widths were between 12–18 Hz FWHM.

### Quantification

The software package LCModel  was used [36,37] for the analysis of the spectra. The quantification algorithm of the LCModel applies linear combinations to calculate the best fit of the experimental spectra to the model spectra. The model spectra were simulated to match the magnetic field strength, type of sequence and sequence parameters used for the data acquisition. The final analysis was performed in the frequency domain with raw data (free induction decay (FID)) as the input. In this study metabolic ratios are used and no absolute concentrations. The ratios are given relative to creatine + phosphocreatine (tCr) as applied by others [21,38,39]. The following 16 metabolites were included in the basis set: alanine (Ala), aspartate (Asp), creatine (Cr), γ-aminobutyric acid (GABA), glucose (Glc), glutamate (Glu), glutamine (Gln), glycerophosphorylcholine (GPC), phosphorylcholine (PCho), *myo*-inositol (m-Ins), lactate (Lac), N-acetylaspartate (NAA), N-acetylaspartylglutamate (NAAG), phosphocreatine (PCr), *scyllo*-inositol, taurine (Tau). Also included are nine simulated macromolecules and lipids.

### Error estimates in the LCModel

In the LCModel the error in the quantification of different metabolites is expressed in percent standard deviation. These values represent the 95% confidence intervals of the estimated concentration values. Table 4 shows the standard error estimates %SD (Cramér-Rao lower bounds, CRLB) for the metabolites included in the analysis. Metabolites which have a %SD >50% are considered to be unreliable and can range from zero to twice the estimated concentration. [40]. If the covariance between two metabolites was high, the sum of the two metabolites was reported (Correlation coefficients >-0.5 and <0.5) [21,41]. The metabolite ratios included in the study were: glutamate (Glu/tCr), glutamine (Gln/tCr), *myo*-inositol (m-Ins/tCr), taurine (Tau/tCr), choline-containing compounds (tCho/tCr) and N-acetylaspartate+N-acetylaspartylglutamate (tNAA/tCr).

**Table 4:** Average Cramér-Rao lower bounds (CRLB) of measured metabolites (± SD).

**Mice type**	**Brain region**	**Gln**	**Glu**	**Tau**	**m-Ins**	**tCho**	**tNAA**
tg	ctx/hipp	33 ± 8%	11 ± 2%	21 ± 7%	16 ± 3%	13 ± 3%	9 ± 2%
tg	str	23 ± 6%	11 ± 4%	11 ± 2%	9 ± 2%	6 ± 1%	8 ± 1%
wt	ctx/hipp	32 ± 8%	9 ± 1%	15 ± 3%	12 ± 2%	9 ± 1%	7 ± 1%
wt	str	20 ± 5%	9 ± 1%	9 ± 1%	7 ± 1%	5 ± 1%	7 ± 1%

Abbreviations: Gln = Glutamine, Glu = Glutamate, Tau = Taurine, m-Ins = *Myo*-inositol, tCho = Choline-containing compounds, tNAA = N-acetylaspartate+N-acetylaspartylglutamate, wt = wild type mice, tg = APP/PS1 transgenic mice, ctx/hipp = parietal cortex/hippocampus, str = striatum, SD = standard deviation.

### Data analysis and statistics

The LCModel data was analyzed using Partial least square discriminant analysis (PLS-DA), a supervised multivariate data analysis method which is part of the SIMCA software (Umetrics AB, Umea, Sweden). PLS-DA analyses were performed using mean centring and unit variance scaling. Mean centring improves the interpretability of the data, by subtracting the average from the individual data for each variable. Large variance variables are more likely to be expressed in modelling than low variance variables. Therefore, unit variance scaling was selected to scale the data appropriately. This scaling method calculates the standard deviation of each variable column. The inverse standard deviation was used as a scaling weight and was multiplied by each column. The results from the PLS-DA analysis were visualized by plotting two components of the model against each other. Each point in the scatter plot represents one individual animal. Each component receives a Q2 value that describes its statistical significance. Q2 values > 0.05 are regarded as statistically significant. Q2 is the fraction of the total variation of the Y's that can be predicted by a component according to cross validation (CV). CV means that a number of parallel models are built. These models differ from each other by leaving out a different individual from the total population each time. The data of the left out animal is then predicted into the respective model which produces a prediction value. A prerequisite for PLS-DA is that two different groups are compared. The prediction values from the two different groups are for one group most correct if close to 1, while for the other group most correct if close to 0. The cut off value for accepting the observation as correctly predicted is 0.5, or in other words <0.5 for one group and >0.5 for the other group. From the respective CV predictions, the sensitivity and specificity of the models can be calculated as follows: Sensitivity, also called the "true positive rate" is defined as the number of true positive predictions divided by the sum of the number of true positive predictions plus the number of false negative predictions. Specificity, also denominated "true negative rate" is defined as the number of true negative predictions divided by the sum of the number of true negative predictions plus the number of false positive predictions.

Further we plotted variables according to their importance for the separation of groups. All the variables received a VIP (variable of importance in the projection) value. The VIP values reflect the importance of the terms in the model. VIP values larger than 1 suggest that the metabolite is over average involved in the separation of groups. Group differences for individual metabolites, also denominated variables, were tested using the paired t-test.

## Abbreviations

AchE: acetylcholine-esterase; AD: Alzheimer's Disease; Ala: alanine; APP: β-amyloid precursor protein; Asp: aspartate; Cr: creatine; CRLB: Cramér-Rao lower bounds; ctx/hipp: fronto-parietal cortex and hippocampus; CV: cross validation; GABA: γ-aminobutyric acid; Glc: glucose; Gln: glutamine; Glu: glutamate; GPC: glycerophosphorylcholine; ^1^H-MRS: Proton magnetic resonance spectroscopy; Lac: lactate; m-Ins: *myo*-inositol; NAA: N-acetylaspartate; NAAG: N-acetylaspartylglutamate; PCho: phosphorylcholine; PCr: phosphocreatine; PLS-DA: Partial least square discriminant analysis; PS1: presenilin 1; RARE: rapid acquisition with relaxation enhancement; str: striatum; Tau: taurine; tCr: creatine + phosphocreatine; TE: echo time; tg: transgenic; TR: repetition time; VIP: variable of importance in the projection; VOI: volumes of interest; wt: wild type.

## Authors' contributions

EW carried out the MRI experiments, designed the MRI protocols, analysed the data, performed the statistical analysis and drafted the manuscript. HR measured the acetylcholine-esterase activity. JÖ helped carry out the MRI experiments. JP supervised the acetylcholine-esterase activity measurements and helped to revise the manuscript. LOW and CS coordinated the MRI study, participated in the MRI protocol design and were the main critical revisers of the manuscript. All authors read and approved the final manuscript.
